# Inhibition of Drp1 Sensitizes Cancer Cells to Cisplatin-Induced Apoptosis through Transcriptional Inhibition of c-FLIP Expression

**DOI:** 10.3390/molecules25245793

**Published:** 2020-12-08

**Authors:** Seon Min Woo, Kyoung-jin Min, Taeg Kyu Kwon

**Affiliations:** 1 Department of Immunology, School of Medicine, Keimyung University, 1095 Dalgubeoldaero, Dalseo-Gu, Daegu 42601, Korea; woosm724@gmail.com (S.M.W.); kjmin@dgmif.re.kr (K.-j.M.); 2 New Drug Development Center, Deagu-Gyeongbuk Medical Innovation Foundation, 80 Chembok-ro, Dong-gu, Daegu 41061, Korea; 3 Center for Forensic Pharmaceutical Science, Keimyung University, 1095 Dalgubeoldaero, Dalseo-Gu, Daegu 42601, Korea

**Keywords:** Mdivi-1, cisplatin, apoptosis, c-FLIP, Drp1

## Abstract

Mitochondrial fragmentation occurs during the apoptosis. Dynamin-related protein 1 (Drp1) acts as an important component in mitochondrial fission machinery and can regulate various biological processes including apoptosis, cell cycle, and proliferation. The present study demonstrates that dysfunction of mitochondrial dynamics plays a pivotal role in cisplatin-induced apoptosis. Inhibiting the mitochondrial fission with the specific inhibitor (Mdivi-1) did not affect apoptotic cell death in low concentrations (<10 μM). Interestingly, mdivi-1 enhanced cisplatin-induced apoptosis in cancer cells, but not in normal cells. Particularly in the presence of mdivi-1, several human cancer cell lines, including renal carcinoma cell line Caki-1, became vulnerable to cisplatin by demonstrating the traits of caspase 3-dependent apoptosis. Combined treatment induced downregulation of c-FLIP expression transcriptionally, and ectopic expression of c-FLIP attenuated combined treatment-induced apoptotic cell death with mdivi-1 plus cisplatin. Collectively, our data provide evidence that mdivi-1 might be a cisplatin sensitizer.

## 1. Introduction

Mitochondrial dynamics, such as fusion, fission, and removal, are complex processes that are precisely regulated by multiple mitochondrial morphology-regulating proteins [1,2]. Modulation of mitochondrial dynamics plays a critical role in the maintenance of mitochondrial functions, resulting in regulation of signals for cell survival or death. This process has been associated with the pathogenesis of neurodegenerative diseases and cancer progression [3,4]. Mitochondrial fission is tightly regulated by cytoplasmic GTPase dynamin-related protein 1 (Drp1). Drp1 is complexed with Fission 1 (Fis1), Mitochondrial fission factor (Mff), or mitochondrial dynamic proteins, and contributes to the constriction and scission of the mitochondrial double membranes [5,6]. Therefore, Drp1 is involved in many biological processes such as mitochondrial biogenesis, cell cycle, proliferation, and apoptosis through the regulation of mitochondrial fission machinery [7,8]. Mitochondrial division inhibitor (mdivi-1) is identified as a small molecule to inhibit Drp1-dependent fission, and potently blocks Bid-activated Bax/Bak-dependent cytochrome *c* release from mitochondria [9]. Mdivi-1 has been shown to protect the heart against ischemia-reperfusion injury and doxorubicin-induced cardiomyopathy by reducing the production of reactive oxygen species [10,11]. Mdivi-1 also protects the macrophages from LPS-induced MAPKs activation, oxidative stress, and cell apoptosis [12]. However, pro-apoptotic effect of mdivi-1 has also been reported. Mdivi-1 enhances death receptor-mediated apoptosis in human ovarian cancer cells [13]. Combined treatment with mdivi-1 and platinum agents induces synergistic apoptosis in platinum-resistant tumor cells [14].

Cisplatin is the first platinum compound and one of the most heavily utilized chemotherapeutic agents for solid tumor malignancies [15]. Cisplatin binds to the purine bases and then induces DNA strand breakage, resulting in cell death [16]. However, the anticancer effect of cisplatin is limited by intrinsic or acquired drug resistance. Cisplatin has been used as a single treatment as well as in combination therapy with other anticancer drugs in various types of cancers. Therefore, identifying chemical reagents to improve the anticancer effect of cisplatin may be an effective strategy for cancer therapy.

This present study investigated the effect of mdivi-1 on cisplatin-induced apoptosis in renal carcinoma cells. We showed that combined treatment with mdivi-1 plus cisplatin can induce apoptotic cell death by reducing the expression of anti-apoptotic c-FLIP protein in renal carcinoma cells.

## 2. Results

### 2.1. Mdivi-1 Does Not Induce Apoptosis in Human Renal Carcinoma Cells

**Figure 1:**
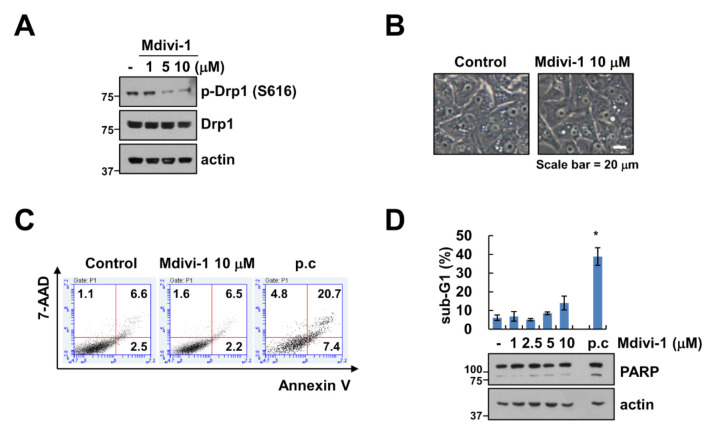
Effect of mdivi-1 on apoptosis in human renal carcinoma cell line Caki-1. (**A**–**D**) Caki-1 cells were treated with 1–10 μM mdivi-1 for 24 h. The cell morphology (**B**) and cell death (**C**) were examined using interference light microscopy and Annexin V/7-AAD staining, respectively. The sub-G1 population and protein expression were detected by flow cytometry (**D**) and Western blotting (**A**,**D**) respectively. Positive control (p.c); TNF-α plus CHX. The values in graph (**D**) represent the mean ± SEM of three independent experiments. * *p* < 0.01 compared to the control.

Since mitochondrial fusion and fission are important for cell function and survival and modulators of mitochondrial division are considered anti-cancer therapeutic agents, we investigated the effects of mitochondrial division inhibitor-1 (mdivi-1), a selective inhibitor to Drp1, on cell death in human renal carcinoma cell line Caki-1. First, we investigated the effect of mdivi-1 on the phosphorylation of Drp1. Mdivi-1 markedly inhibited the phosphorylation of serine residues 616 of Drp1 in a dose-dependent manner (Figure 1A). Next, Caki-1 cells were treated with vehicle or 10 μM mdivi-1 for 24 h; cell morphology and apoptosis were then measured using microscopy and 7-AAD and annexin V staining, respectively. As shown in Figure 1B,C, mdivi-1 did not induce cell morphology change and apoptosis. In addition, mdivi-1 did not increase sub-G1 population and PARP cleavage, which is an apoptosis hall marker, whereas TNF-α plus cycloheximide increased sub-G1 population and cleavage of PARP (Figure 1D). These results indicate that although mdivi-1 inhibits phosphorylation of Drp1 (Ser616), mdivi-1 treatment did not induce apoptosis in human renal carcinoma cell line Caki-1.

### 2.2. Mdivi-1 Enhances Cisplatin-Induced Apoptosis

**Figure 2:**
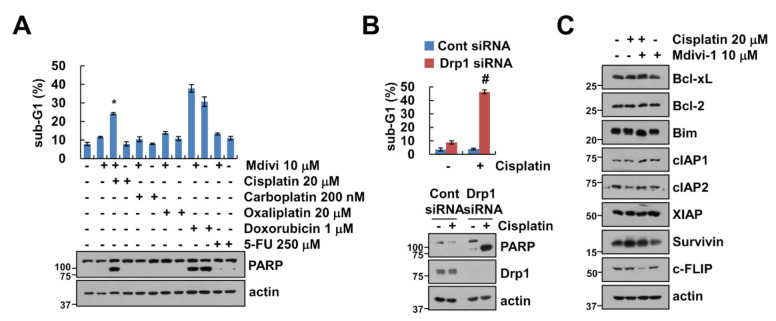
Mdivi-1 increases cisplatin-mediated apoptosis. (**A**) Caki-1 cells were treated with a combination of 20 μM cisplatin, 200 nM carboplatin, 20 μM oxaliplatin, 1 μM doxorubicin, and 250 μM 5-FU in the presence or absence of 10 μM mdivi-1 for 24 h. (**B**) Caki-1 cells were transfected with Cont or Drp1 siRNA and were treated with 20 μM cisplatin for 24 h. The sub-G1 population and protein expression were detected by flow cytometry (**A**,**B**) and Western blotting (**A**–**C**), respectively. The values in graph (**A**,**B**) represent the mean ± SEM of three independent experiments. * *p* < 0.01 compared to the control. # *p* < 0.01 compared to cisplatin in Drp1 siRNA.

Next, we examined whether mdivi-1 enhanced anti-cancer drugs-induced apoptosis. We selected 10 μM mdivi-1 to sensitize sub-toxic dosage of anti-cancer drugs-induced apoptosis. Among anti-cancer drugs, mdivi-1 markedly enhanced cisplatin-induced sub-G1 population and PARP cleavage. To further confirm significance of Drp1 inhibition, we investigated the effect of Drp1 knock-down (KD). Sub-lethal dose of cisplatin also induced apoptosis and PARP cleavage in Drp1 KD cells (Figure 2B). Therefore, we focused on the molecular mechanism of apoptosis induced by combined treatment with cisplatin and mdivi-1. We analyzed the expression of apoptosis-related proteins. As shown in Figure 2C, the expression levels of tested apoptosis-related proteins were not changed by the combined treatment. However, mdivi-1 plus cisplatin induced the downregulation of c-FLIP protein expression. Therefore, these results indicate that mdivi-1 plus cisplatin induced the downregulation of c-FLIP expression.

### 2.3. Combined Treatment with Mdivi-1 and Cisplatin Induces Activation of Caspase in Renal Carcinoma Cell Line Caki-1

**Figure 3:**
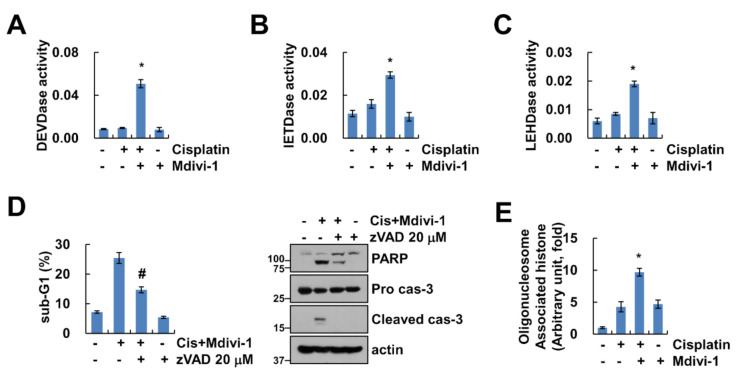
Combined treatment of mdivi-1 plus cisplatin induces caspase-dependent cancer cell death. (**A**–**C**) Caki-1 cells were treated with 10 μM mdivi-1 alone, 20 μM cisplatin alone, or mdivi-1 plus cisplatin for 24 h, and measured DEVDase (caspase-3, **A**), IETDase (caspase-8, **B**) or LEHDase (caspase-9, **C**) activity. (**D**) Caki-1 cells were treated with 10 μM mdivi-1 plus 20 μM cisplatin in the presence or absence of 20 μM z-VAD for 24 h. The sub-G1 population and protein expression were detected by flow cytometry and Western blotting, respectively. (**E**) Caki-1 cells were treated with 10 μM mdivi-1 alone, 20 μM cisplatin alone, or mdivi-1 plus cisplatin for 24 h, and detected DNA fragmentation using detection kit. The values in graph (**A**–**E**) represent the mean ± SEM of three independent experiments. * *p* < 0.01 compared to the control. # *p* < 0.01 compared to the mdivi-1 plus cisplatin.

We performed further analysis for determination of whether combined treatment with mdivi-1 and cisplatin affects activation of caspases, key executioners of apoptosis. As shown in Figure 3A–C, mdivi-1 plus cisplatin increased caspase-3 (DEVDase), -8 (IETDase) and -9 (LEHDase) activity. To investigate whether the activation of caspases plays a crucial role in the apoptotic pathway induced by combined treatment, Caki-1 cells were pretreated with potential pan-caspase inhibitor, z-VAD-fmk, followed by treatment with mdivi-1 plus cisplatin for 24 h. As shown in Figure 3D, z-VAD markedly inhibited cleavage of PARP and activation of caspase-3 in mdivi-1 plus cisplatin-treated cells (Figure 3D). In addition, combined treatment markedly induced cytoplasmic histone-associated DNA fragments (Figure 3E). Therefore, these data indicate that combined treatment with mdivi-1 plus cisplatin induces caspase-dependent apoptosis in human renal carcinoma cell line Caki-1.

### 2.4. Downregulation of c-FLIP Plays a Critical Role in Mdivi-1 Plus Cisplatin-Induced Apoptosis

**Figure 4:**
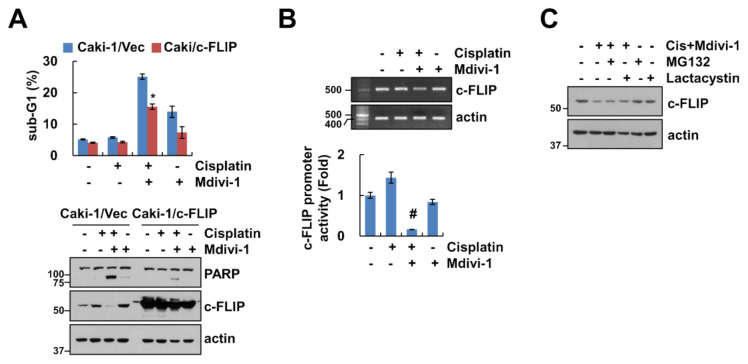
Combined treatment of mdivi-1 plus cisplatin inhibits c-FLIP expression at the transcription level. (**A**) Vector cells and c-FLIP-overexpressing cells were treated with 10 μM mdivi-1 alone, 20 μM cisplatin alone, or mdivi-1 plus cisplatin for 24 h. (**B**) Caki-1 cells were treated with 10 μM mdivi-1 alone, 20 μM cisplatin alone, or mdivi-1 plus cisplatin for 24 h. The mRNA expression and promoter activity were detected using reverse transcription polymerase chain reaction (RT-PCR) and luciferase activity kits, respectively. (**C**) Caki-1 cells were treated with 10 μM mdivi-1 plus 20 μM cisplatin in the presence or absence of 0.5 μM MG132 or 2.5 μM lactacystin for 24 h. The sub-G1 population and protein expression were detected by flow cytometry (**A**) and Western blotting (**A**,**C**), respectively. The values in graph (**A**,**B**) represent the mean ± SEM of three independent experiments. * *p* < 0.01 compared to the mdivi-1 plus cisplatin in Caki-1/Vec. # *p* < 0.01 compared to the control.

We investigated whether c-FLIP plays a critical role in apoptosis in mdivi-1 plus cisplatin-treated cells. Ectopic expression of c-FLIP markedly inhibited mdivi-1 plus cisplatin-induced sub-G1 population and cleavage of PARP (Figure 4A). To further investigate the molecular mechanisms underlying the downregulation of c-FLIP in combined treatment, we analyzed the transcriptional regulation of c-FLIP. The expression levels of c-FLIP mRNA were downregulated by combined treatment (Figure 4B). Also, combined treatment of mdivi-1 and cisplatin inhibited c-FLIP promoter activity (Figure 4B). Since c-FLIP expression is also regulated by the ubiquitin–proteasome pathway, we examined whether combined treatment with mdivi-1 and cisplatin can modulate c-FLIP at the post-translational level. Proteasome inhibitors (MG132 and lactacystin) did not rescue combined treatment-induced downregulation of c-FLIP expression (Figure 4C). Therefore, these results suggest that mdivi-1 plus cisplatin induced downregulation of c-FLIP at the transcriptional levels, which is critical for enhancement of apoptosis.

### 2.5. Combined Treatment with Mdivi-1 plus Cisplatin Induces Apoptosis in Other Cancer Cells, but Not Normal Cells

**Figure 5:**
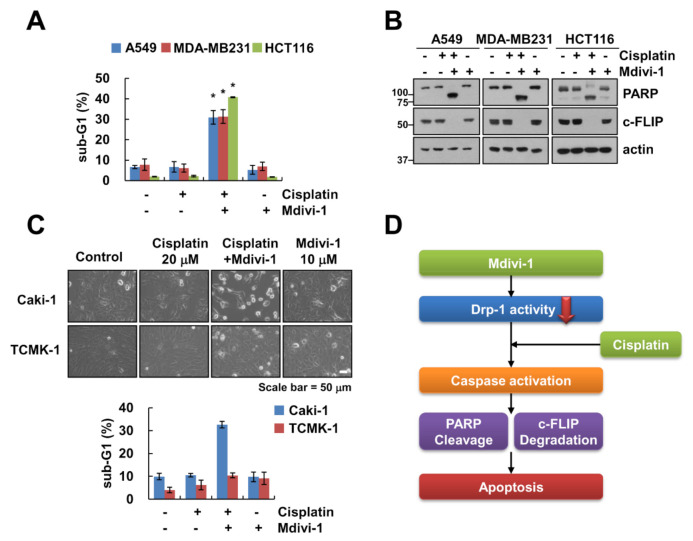
Effect of combined treatment of mdivi-1 and cisplatin on apoptosis in other cancer and normal cells. (**A**–**C**) Indicated cancer cells (**A**,**B**) or normal renal TCMK-1 cells (**C**) were treated with 10 μM mdivi-1 alone, 20 μM cisplatin alone, or mdivi-1 plus cisplatin for 24 h. (**D**) The scheme indicating the mechanism of combined treatment of mdivi-1 and cisplatin. The sub-G1 population and protein expression were detected by flow cytometry (**A**,**C**) and Western blotting (**B**), respectively. The cell morphology was examined using interference light microscopy (**C**). The values in graph (**A**,**C**) represent the mean ± SEM of three independent experiments. * *p* < 0.01 compared to the control.

To investigate the effect of mdivi-1 on cisplatin-induced apoptosis in other cancer cells, we combined treatment with mdivi-1 and cisplatin in lung carcinoma A549 cells, breast carcinoma MDA-MB231 cells, and colon carcinoma HCT116 cells. Combined treatment with mdivi-1 plus cisplatin markedly induced apoptosis and PARP cleavage in other cancer cells (Figure 5A,B). As shown in Figure 5C, mdivi-1 or cisplatin alone did not affect cell morphology change, whereas combination treatment increased population of detached cells and apoptotic morphology. In contrast, combined treatment with mdivi-1 plus cisplatin had no effect on morphological change and induction of sub-G1 population in normal mouse kidney cells (TCMK-1) (Figure 5C). These data indicate that combined treatment with mdivi-1 plus cisplatin might induce apoptosis in cancer cells, but not normal cells.

## 3. Discussion

Mdivi-1 is known as an inhibitor of mitochondrial division target for Drp1 [9], and has therapeutic effects for stroke, myocardial infarction, and neurodegenerative diseases [17]. However, there are no reports about the anticancer effect on renal carcinoma cells. In this study, we demonstrated that mdivi-1 augmented cisplatin-induced apoptosis in renal cancer cells, but not normal cells. We found that the mechanism of mdivi-1-mediated cisplatin sensitization is associated with downregulation of c-FLIP. These data supported the hypothesis that mdivi-1 could react as an attractive agent for cisplatin-sensitization (Figure 5D).

Previous studies have shown that hypoxia-induced reactive oxygen species (ROS) stimulated an increase in mitochondrial fission and cisplatin resistance. Therefore, inhibition of Drp1 by mdivi-1 treatment or knockdown of Drp1 by siRNA enhanced cisplatin sensitivity of ovarian cancer cells under hypoxia [18]. Tusskorn et al. reported that mdivi-1 sensitized cholangiocarcinoma cells to cytotoxicity of cisplatin in association with increases of oxidative stress and autophagosomes [19]. Interestingly, Wang et al. have demonstrated that mdivi-1 enhances death ligand-mediated apoptosis independent of Drp1. Combined treatment with mdivi-1 plus tumor necrosis factor-related apoptosis-inducing ligand (TRAIL) induced a similar pattern in the increase of Annexin V-positive apoptotic cells in both Drp1 wild-type and Drp1 knockout cells [13]. However, multiple studies have reported that inhibition of Drp1-dependent mitochondrial division delays and partially inhibits apoptosis [20,21,22]. This contradiction is believed to be due to the difference in the cell contexts and stimuli. Therefore, it needs to be further studied to identify the molecular mechanisms of Drp1 inhibition or mdivi-1-mediated apoptosis.

We observed that combined treatment with mdivi-1 and cisplatin induced downregulation of c-FLIP expression (Figure 2C). c-FLIP is a major anti-apoptotic protein and is involved in death receptor-mediated apoptosis and chemotherapeutic drug resistance in cancer cells [23]. To investigate the role of c-FLIP downregulation in combined treatment with mdivi-1 plus cisplatin-induced apoptosis of Caki-1 cells, we used c-FLIP overexpressing cells. Overexpression of c-FLIP attenuated combined treatment-induced apoptosis (Figure 4A). The expression of levels of c-FLIP protein is regulated by multiple mechanisms such as transcriptional, translational, and post-translational regulation [24,25,26]. Our data indicate that combined treatment-induced c-FLIP downregulation is shown to be related to the transcriptional regulation (Figure 4B). Proteasome inhibitor, MG132, and lactacystin, had no effect on the combined treatment-induced c-FLIP downregulation (Figure 4C). Therefore, the mechanism of combined treatment-mediated c-FLIP downregulation at the transcriptional levels requires further investigation. Further investigation of the functional mechanism of mdivi-1 in enhancing cisplatin-mediated apoptotic cell death may lead to a better understanding of cisplatin resistance in multiple cancer cells. Further in vivo studies are needed to realize the development of novel therapeutic strategies against cancer cells.

Collectively, these results suggest that combined treatment with mdivi-1 and cisplatin is highly effective in inducing apoptosis in renal carcinoma and other carcinoma cell lines, but not in normal cells. Therefore, mdivi-1 may be effectively used as an adjuvant or sensitizer of cisplatin.

## 4. Materials and Methods

### 4.1. Cell Lines

All cancer cells (Caki-1, A549, MDA-MB231, and HCT116) and TCMK-1 cells were obtained from American Type Culture Collection (Manassas, VA, USA). Cells were grown in an appropriate medium supplemented with 10% fetal bovine serum (FBS) (Welgene, Gyeongsan, Korea), 1% penicillin–streptomycin and 100 μg/mL gentamycin (Thermo Fisher Scientific, Waltham, MA, USA). For constructing stable cell lines, Caki-1 cells were transfected using Lipofectamine^TM^ 2000 (Invitrogen, Carlsbad, CA, USA) with the pcDNA3.1(+)/c-FLIP or pcDNA3.1(+) vector plasmids. These plasmids were transduced for 24 h and cells were selected by 700 μg/mL G418 (Invitrogen, Carlsbad, CA, USA). For knockdown of the gene by siRNA, Lipofectamine^®^ RNAiMAX Reagent (Invitrogen, Carlsbad, CA, USA) was used in Caki-1 cells. Immunoblot analysis was performed to examine protein expression [27].

### 4.2. Reagents and Antibodies

Sigma Chemical Co. provided mdivi-1, MG132 an anti-β-actin (St. Louis, MO, USA), and an R&D system supplied z-VAD-fmk and anti-survivin (Minneapolis, MN, USA). Enzo Life Sciences provided lactacystin, anti-pro-caspase-3, and anti-c-FLIP (San Diego, CA, USA). Anti-PARP, anti-cleaved caspase-3, and anti-Bcl-xL were supplied from Cell Signaling Technology (Beverly, MA, USA). Anti-Bim and anti-XIAP were provided from BD Biosciences (San Jose, CA, USA). Anti-Mcl-1, anti-Bcl-2, and anti-cIAP2 were obtained from Santa Cruz Biotechnology (St. Louis, MO, USA).

### 4.3. Annexin V and 7-AAD Staining

FITC-conjugated Annexin V and 7-aminoactinomycin D (7-AAD) (BD Pharmingen, San Jose, CA, USA) were used to estimate cell death mode. Cells were washed in cold phosphate buffered saline (PBS) and resuspended in binding buffer. We added Annexin V-FITC and 7-AAD into the suspended cells, and then incubated for 15 min at room temperature in the dark. The cell death population was detected by a BD Accuri™ C6 flow cytometer (BD Biosciences, San Jose, CA, USA).

### 4.4. FACS Analysis

For apoptosis analysis, cells were harvested and suspended in 100 μL of phosphate-buffered saline and added to 200 μL of 95% ethanol. After that, cells were incubated in 1.12% sodium citrate buffer containing RNase at 37 °C for 30 min, added to 50 μg/mL propidium iodide, and analyzed using a BD Accuri™ C6 flow cytometer (BD Biosciences, San Jose, CA, USA).

### 4.5. Western Blotting

Cells were lysed in radioimmunoprecipitation assay (RIPA) lysis buffer (20 mM HEPES and 0.5% Triton X-100, pH 7.6) and separated by 10% SDS-PAGE [28]. Proteins were transferred to nitrocellulose membranes (GE Healthcare Life Science, Pittsburgh, PA, USA) and detected using an Immobilon Western Chemiluminescent HRP Substrate (EMD Millipore, Darmstadt, Germany) for analysis protein expression.

### 4.6. DNA Fragmentation and Caspase Activity Assay

Caki-1 cells were treated with mdivi-1 alone, cisplain alone or combined treatments. To measure DNA fragmentation, we used cell death detection ELISA plus kit (Boehringer Mannheim, Indianapolis, IN, USA) according to the manufacturer’s recommendations. For caspase activity assay, cells were harvested and incubated with a reaction buffer containing acetyl-Asp-Glu-Val-Asp *p*-nitroanilide (Ac-DEVD-pNA), acetyl-Ile-Glu-Thr-Asp-*p*-nitroanilide (Ac-IETD-pNA), or Ac-Leu-Glu-His-Asp-*p*-nitroaniline (Ac-LEHD-pNA) substrate, as previously described [29].

### 4.7. Reverse Transcription Polymerase Chain Reaction (RT-PCR)

Total RNA was isolated with TriZol reagent (Life Technologies, Gaithersburg, MD, USA), and prepared cDNA using M-MLV reverse transcriptase (Gibco-BRL, Gaithersburg, MD, USA). For PCR, we used Blend Taq DNA polymerase (Toyobo, Osaka, Japan) with primers targeting c-FLIP and actin. The used primers were referred to in previous studies [30]. The following primers were used: human c-FLIP and actin; c-FLIP (58 °C) (forward) 5′-CGG ACT ATA GAG TGC TGA TGG-3′ and (reverse) 5′-GAT TAT CAG GCA GAT TCC TAG-3′; and actin (56 °C) (forward) 5′- GGC ATC GTC ACC AAC TGG GAC-3′ and (reverse) 5′-CGA TTT CCC GCT CGG CCG TGG-3′.

### 4.8. Luciferase Activity Assay

The c-FLIP promoter-constructs transfected into the cells using Lipofectamine™2000 (Invitrogen, Carlsbad, CA, USA). After that, cells were collected and harvested in a lysis buffer [31]. The supernatants were used to measure the luciferase activity according to the manufacturer’s instructions (Promega, Madison, WI, USA).

### 4.9. Statistical Analysis

The data were analyzed using a one-way ANOVA and post-hoc comparisons (Student–Newman–Keuls) using the Statistical Package for Social Sciences 22.0 software (SPSS Inc., Chicago, IL, USA).

## 5. Conclusions

Mdivi-1, mitochondria division inhibitor, sensitizes cancer cells to cisplatin-induced apoptosis through c-FLIP downregulation. We demonstrated that combined treatment with mdivi-1 plus cisplatin induced downregulation of c-FLIP in the transcriptional level. Therefore, mdivi-1 may be effectively used as an adjuvant or sensitizer of cisplatin.
